# Microglia activation is essential for BMP7-mediated retinal reactive gliosis

**DOI:** 10.1186/s12974-017-0855-0

**Published:** 2017-04-05

**Authors:** Subramanian Dharmarajan, Debra L. Fisk, Christine M. Sorenson, Nader Sheibani, Teri L. Belecky-Adams

**Affiliations:** 1 Department of Biology, Indiana University-Purdue University Indianapolis, 723 W Michigan St, SL306, Indianapolis, IN 46202 USA; 2 Center for Developmental and Regenerative Biology, Indiana University-Purdue University Indianapolis, 723 W Michigan St, Indianapolis, IN 46202 USA; 3 Department of Ophthalmology and Visual Sciences, University of Wisconsin School of Medicine and Public Health, 1111 Highland Avenue, 9453 WIMR, Madison, WI 53705 USA; 4 Department of Pediatrics, University of Wisconsin School of Medicine and Public Health, 1111 Highland Avenue, 9453 WIMR, Madison, WI 53705 USA

**Keywords:** Microglia, Reactive gliosis, BMP7, Retina, Müller glia, Retinal astrocytes

## Abstract

**Background:**

Our previous studies have shown that BMP7 is able to trigger activation of retinal macroglia. However, these studies showed the responsiveness of Müller glial cells and retinal astrocytes in vitro was attenuated in comparison to those in vivo, indicating other retinal cell types may be mediating the response of the macroglial cells to BMP7. In this study, we test the hypothesis that BMP7-mediated gliosis is the result of inflammatory signaling from retinal microglia.

**Methods:**

Adult mice were injected intravitreally with BMP7 and eyes harvested 1, 3, or 7 days postinjection. Some mice were treated with PLX5622 (PLX) to ablate microglia and were subsequently injected with control or BMP7. Processed tissue was analyzed via immunofluorescence, RT-qPCR, or ELISA. In addition, cultures of retinal microglia were treated with vehicle, lipopolysaccharide, or BMP7 to determine the effects of BMP7-isolated cells.

**Results:**

Mice injected with BMP7 showed regulation of various inflammatory markers at the RNA level, as well as changes in microglial morphology. Isolated retinal microglia also showed an upregulation of BMP-signaling components following treatment. In vitro treatment of retinal astrocytes with conditioned media from activated microglia upregulated RNA levels of gliosis markers. In the absence of microglia, the mouse retina showed a subdued gliosis and inflammatory response when exposed to BMP7.

**Conclusions:**

Gliosis resulting from BMP7 is mediated through an inflammatory response from retinal microglia.

**Electronic supplementary material:**

The online version of this article (doi:10.1186/s12974-017-0855-0) contains supplementary material, which is available to authorized users.

## Background

The mammalian retina consists of at least two distinct glial populations: the macroglia, which includes Müller glia and retinal astrocytes, and the microglia. The Müller glia are the primary glial cells found in the retina, having their nucleus in the inner nuclear layer (INL) with processes extending from the inner limiting membrane at the vitreal border to the outer limiting membrane at the base of the photoreceptor inner segments [1]. Retinal astrocytes migrate into the retina from the optic nerve and reside in the nerve fiber layer [2]. The microglia are the resident macrophages found scattered through all the retinal layers [3]. The retina of some species also contain oligodendrocytes and another glial-like cell type, known as the non-astrocytic inner retinal glia-like (NIRG) cells, that reside in the INL of the chick retina [4, 5].

Müller glial cells and retinal astrocytes are essential for maintaining retinal homeostasis. Any injury or disease leading to retinal damage or disruption of the homeostasis triggers the glial cells to become active, a response termed reactive gliosis. Reactive gliosis has been observed in all retinal disease and injury models including glaucoma, age-related macular degeneration, and diabetic retinopathy [6–9]. Reactive gliosis is characterized by hypertrophy, altered function brought about by changes in expression of proteins such as glutamine synthetase (GS), S100-β, extracellular matrix proteins, chondroitin sulfate proteoglycans (CSPG), matrix metalloproteinases (MMP), and an increase in growth factors such as ciliary neurotrophic factor (CNTF), leukemia inhibitory factor (LIF), and vascular endothelial growth factor (VEGF) [10, 11]. Multiple factors can trigger gliosis, including the bone morphogenetic proteins (BMPs) [12–14]. Recent evidence from the Belecky-Adams laboratory showed that BMP7 triggered gliosis in both the Müller glia and astrocytes of the mouse retina; however, the mechanism by which BMP7 triggers gliosis is unknown [11].

The BMPs are growth factors that belong to the transforming growth factor beta (TGF-β) superfamily. BMP signaling is initiated following the binding of the ligand to serine threonine kinase receptors. This leads to the activation of the receptors and the subsequent phosphorylation and activation of downstream signaling components. In the canonical pathway, the BMP signals by phosphorylation and activation of downstream receptor SMADs (RSMADs). The RSMADs form a dimer with the co-SMAD (SMAD4) and are shuttled to the nucleus to regulate transcription. BMP can also mediate the activation of a non-canonical pathway referred to as the BMP mitogen-activated protein kinase pathway (BMP-MAPK). In the BMP-MAPK pathway, the receptors recruit the X-linked inhibitor of apoptosis (XIAP) to a complex containing TAB1 and TAK1, thereby activating TAK1. TAK1 then activates downstream kinases, eventually activating NF-κB, p38, and JNK MAPKs [15, 16]. In the CNS, BMP regulation has been observed in various diseases and injury models, such as spinal cord injuries, axonal damage, and ischemia [14, 17, 18]. In the retina, upregulation of BMPs and their signaling components are observed in the photo-damaged retina injury model and in diabetic retinopathy [19–21].

Microglia are the innate immune cells of the retina. In their resting state, the microglia act as sentinels, extending their processes throughout the retina. In the mouse retina, the microglia are initially found in the ganglion cell layer, entering the retina from the ciliary marginal zone and vitreous. By postnatal day 7, the microglia spread to the rest of the retinal layers, finally resting in the plexiform layers [22]. Upon receiving signals from injured or dying cells, the microglial cells become activated: they retract their processes, undergo an increase in cellular area, become amoeboid in shape, and migrate to the area of injury or disease to phagocytize cellular debris and metabolic products [23, 24]. Stimuli such as neuronal loss or damage, inflammation, and nerve degeneration activate the microglia into a motile effector cell with altered morphological characteristics [25, 26].

Microglial activation has been observed in all retinal diseases, including diabetic retinopathy, age-related macular degeneration, glaucoma, and models of retinal pathologies. In addition to the morphological changes following activation, microglia also induce a change in production of various cytokines such as interleukin 1 beta (IL-1β), IL-6, and interferon gamma (IFN-γ), chemokines such as RANTES, MCP1, growth factors such as colony stimulating factor (CSF) and VEGF, and various scavenger receptors and antigen-presenting molecules such as the scavenger receptor A (SR-A) and major histocompatibility complex (MHC) [3, 27]. Furthermore, research has revealed that activated microglia can be further classified into the following phenotypes: the M1 or proinflammatory phenotype and the M2 or the anti-inflammatory phenotype [28, 29]. Polarization to the M1 phenotype, following exposure to factors such as lipopolysaccharide (LPS) and IFN-γ, the microglia upregulate proinflammatory factors such as IL-1β, tumor necrosis factor alpha (TNF-α), inducible nitric oxide synthase (iNOS), SRs, and MHC-II [30, 31]. The M2 phenotype plays a role in the resolution of the inflammation and tissue remodeling. This phenotype is induced by factors such as IL-4 and IL-10 or through the maturation of the M1 cells. This phenotype was characterized by an upregulation of markers such as arginase-1 (*Arg-1*) and mannose receptor (*Mr*), cytokines such as IL-10 and IL-13, and growth factors such as TGF-β and VEGF [30, 32].

Signals from neurons and macroglia, such as fractalkine, neurotransmitters, and neurotrophins help keep the glial population in the quiescent state [6, 33]. Activation of the glial cells has been found to be mediated by similar stimuli in vitro and in retinal disease models in vivo [6, 25, 34–36]. Cytokines and other inflammatory markers such as TNF-α, iNOS, CNTF, and LIF are not only regulated during gliosis but are also factors known to act on the glial cells and regulate gliosis [20, 37, 38]. Activated microglia are known to regulate Müller cell activity directly, regulating cell morphology, proliferation, and gene expression [26, 39]. Activated microglia can also regulate the generation of Müller glia-derived progenitors [40]. Here, we provide evidence that supports the hypothesis that BMP7 indirectly triggers gliosis by activating the proinflammatory state of retinal microglia.

## Methods

### Cell culture

Mouse retinal astrocytes were isolated in the Sheibani lab and maintained as previously described in [11, 41]. Microglial cells were isolated from retinas of newborn (P0-P4) immortomouse back crossed into C57BL/6J as described in [42] with some modifications. Briefly, the retinas were placed in a solution of Trypsin/EDTA (5 ml; 0.25% trypsin and 1 mM EDTA; Thermo Scientific) and incubated at 37 °C for 5 min. Following incubation, the samples were triturated by pipette, and 5 ml of DMEM with 10% FBS was added to stop trypsin activity. The digested tissue was centrifuged for 5 min at 400 × *g* at room temperature, the supernatant was carefully aspirated, and the pellet was re-suspended in the microglia medium [a 1:1 mixture of DMEM: F12 (Thermo Scientific) containing 10% FBS and 44 U/ml of interferon-γ (R&D Systems, Minneapolis, MN)], plated on a single well of a 6-well plate, and incubated in a tissue culture incubator at 33 °C and 5% CO_2_. The cells were allowed to grow for 1-2 weeks and fed every 3-4 days until nearly confluent. The medium was then removed from the plate and rinsed with PBS containing 0.04% EDTA. The plate was then incubated with 2 ml of PBS containing 0.04% EDTA and placed on a multipurpose rotator at 100 rpm at room temperature for 20–30 min. The supernatant was collected in a 15-ml tube containing 3 ml of DMEM with 10% FBS and centrifuged at 400 × *g* for 5 min. The detached cells were then re-plated in the microglia medium, allowed to reach confluence, and expanded into 60-mm dishes. The purity of the microglial cultures was inspected by immunocytochemical staining and flowcytometric analysis for F4/80 (eBiosciences; San Diego, CA) and keratin sulfate (Seikagaku Corporation; Jersey City, NJ). The purity of culture was nearly 95% using FACS and immunostaining analysis. Astrocytes and microglia were grown in tissue culture dishes (BD Falcon) in an incubator with 5% CO_2_ at 33 °C and passaged every 5–7 days using trypsin-EDTA, and the medium changed every 3–4 days. Cells were treated with 1 μl/ml vehicle (4 mM HCL with 0.1% BSA), 100 ng/ml of mouse bone morphogenetic protein 7 (BMP7; R&D systems), 300 ng/ml mouse interferon-gamma (IFN-γ; R&D systems), or 100 ng/ml LPS (Sigma). Medium from microglial cells incubated with BMP7 or vehicle (conditioned medium) for 24 h was used to treat retinal astrocytes. The conditioned medium was added to the retinal astrocytes medium at 25% concentration in the presence of DMSO or 2.5 μM ALK2/ALK3/ALK6 inhibitor LDN193189 [43]. The retinal astrocytes were allowed to grow for 24 h; after which, cells were harvested for RNA isolation and RT-qPCR analysis.

### Experimental groups

Experiments were carried out in 4–8 weeks old male C57BL/6J. All procedures were in accordance with the guidelines set by the Institutional Animal Care and Use Committee (IACUC) at the school of science IUPUI (protocol number SC230R). For BMP7 injection studies, *n* = 8 mice were used with the left eye injected with the vehicle and the right eye injected with BMP7. For the PLX studies, two groups of mice were considered, the age-matched control chow group (*n* = 12) and the PLX group (*n* = 12), kept on PLX chow diet. For the PLX BMP7 injection studies, two groups of mice were considered, the age-matched control group (*n* = 12) and the PLX group (*n* = 12), kept on the PLX diet. Both the groups were injected with the vehicle control in the left eye and BMP7 in the right eye. P30 VE-YFP mice (*n* = 3), which express YFP in endothelial cells, generated by crossing a line of mice containing an enhanced yellow fluorescent protein (YFP) with a floxed stop sequence upstream of the YFP (B6.129X1-Gt(ROSA)26Sor^tm1(EYFP)Cos^/J; strain number 006148 Jackson laboratory) [44] with the VE-cadherin-cre line (B6.FVB-Tg(Cdh5-cre)7Mlia/J; stock number 006137 Jackson laboratory) [45], were used for immunofluorescence experiments determining PU.1 co-localization in the retina.

### Intraocular injections

Postnatal day 30 (P30), C57BL/6J mice were anesthetized with ketamine and xylazine cocktail. Mice were injected intravitreally with 1 μl of vehicle (4 mM HCL with 0.1%BSA) or 1 μl BMP7 (20 ng/μl) as previously stated in [11]. Intraocular injections were performed using a manual microsyringe (World Precision Instruments) and pulled glass micropipettes.

### Microglia ablation

C57BL/6J mice were kept on chow feed containing 1200 ppm PLX5622 (PLX; Plexxikon Inc.) for up to 21 days, starting at P30. Eyes were harvested at 7, 14, and 21 days following start of the PLX diet for assessment of loss of microglia. The control mice were kept on the control chow supplied by Plexxikon Inc. To determine if loss of microglial cells affected BMP7-mediated gliosis, mice were maintained on PLX chow for the entirety of the experiment. In some animals, eyes were injected with 1 μl vehicle (4 mM HCL with 0.1%BSA) or 1 μl (20 ng/μl) BMP7 14 days following treatment with PLX, and eyes were harvested and processed 3 and 7 days postinjection.

### Tissue processing

Eyes from euthanized C57BL/6J mice were enucleated, washed in PBS, and either fixed in 4% paraformaldehyde (PFA) for immunofluorescence (IF) or dissected to isolate the retina for preparation of RNA and/or protein. For IF analysis, enucleated eyes were washed and fixed in 4% PFA, incubated in ascending series of sucrose, and frozen in a sucrose OCT solution as previously described [11]. Thick sections (12 μm) were cut using Leica CM3050S cryostat onto Superfrost Plus slides (ThermoScientific) and stored at −80 °C until use. Retinas from enucleated eyes were isolated as previously described [11]. Isolated retinas were immediately processed for RNA isolation using RNeasy kit (Qiagen).

### RT-qPCR

Reverse transcriptase-quantitative polymerase chain reaction (RT-qPCR) was performed to detect changes in markers associated with gliosis and inflammation as previously described [11]. The primers for RT-qPCR analysis are listed in Table 1 of [11]. Included in this table is the accession number of each gene, the sequence of each primer, product length, and calculated efficiency of each primer. Primers used for assessing changes in inflammation are listed in Table 1. RT-qPCR was performed using SYBR green master mix (Roche) with the reactions carried out in the LighCycler480 system (Roche). The change in RNA levels was measured using the 2 ^−ΔΔCt^ method, where Ct is the crossing threshold/crossing point (Cp) value. Relative RNA levels were calculated using the geometric means from the Ct value derived from three housekeeping genes: *β- 2 Microglobulin* (*B2m*), *succinate dehydrogenase complex subunit A (Sdha)*, and *signal recognition particle 14 kDa (Srp14)*. A no template control was also tested for each marker.

**Table 1:** The primers used for qPCR analysis

Gene	Primer	Sequence	Product length
*Gm-Csf*	Forward	AGTCGTCTCTAACGAGTTCTCC	178
	Reverse	AACTTGTGTTTCACAGTCCGTT	
*Csf1*	Forward	ACCAAGAACTGCAACAACAGC	91
	Reverse	GGGTGGCTTTAGGGTACAGG	
*Inf-α*	Forward	CAAGCCATCCCTGTCCTGAG	131
	Reverse	TCATTGAGCTGCTGGTGGAG	
*Inf-γ*	Forward	CAACAGCAAGGCGAAAAAGGA	90
	Reverse	AGCTCATTGAATGCTTGGCG	
*Il-1β*	Forward	TGTCTGAAGCAGCTATGGCAA	141
	Reverse	GACAGCCCAGGTCAAAGGTT	
*Il6*	Forward	ACTTCACAAGTCGGAGGCTT	111
	Reverse	TGCAAGTGCATCATCGTTGT	
*VEGF*	Forward	ACTGGACCCTGGCTTTACTG	74
	Reverse	CTCTCCTTCTGTCGTGGGTG	
*Tnf-α*	Forward	TAGCCCACGTCGTAGCAAAC	136
	Reverse	ACAAGGTACAACCCATCGGC	
*Ccl5*	Forward	TGCCCACGTCAAGGAGTATTT	111
	Reverse	ACCCACTTCTTCTCTGGGTTG	
*Thbs1*	Forward	GCCACAGTTCCTGATGGTGA	149
	Reverse	TTGAGGCTGTCACAGGAACG	
*Thbs2*	Forward	GGGAGGACTCAGACCTGGAT	105
	Reverse	CGGAATTTGGCAGTTTGGGG	
*Cd45*	Forward	TGACCATGGGTTTGTGGCTC	134
	Reverse	TTGAGGCAGAAGAAGGGCAT	
*Cd68*	Forward	AAGGGGGCTCTTGGGAACTA	139
	Reverse	AAGCCCTCTTTAAGCCCCAC	
*Iba1*	Forward	ACGAACCCTCTGATGTGGTC	118
	Reverse	TGAGGAGGACTGGCTGACTT	
*Irf8*	Forward	CGGATATGCCGCCTATGACA	73
	Reverse	CTTGCCCCCGTAGTAGAAGC	
*Sdha*	Forward	GGACAGGCCACTCACTCTTAC	130
	Reverse	CACAGTGCAATGACACCACG	
*Srp14*	Forward	CCTCGAGCCCGCAGAAAA	134
	Reverse	CGTCCATGTTGGCTCTCAGT	

### Immunofluorescence

Frozen tissue sections were labeled as previously described [11]. Antigen retrieval was performed by using 1% sodium dodecyl sulfate (SDS) in 0.01 M PBS (5 min at room temperature) or by heat antigen retrieval method. Briefly, sections were washed with 1× PBS, postfixed with 4% PFA, and permeabilized with methanol. Sections were then incubated in 10 mM sodium citrate buffer at 65 °C for 45 min, allowed to cool at room temperature (RT) for 20 min, rinsed in deionized (DI) water 3×, and washed in PBS once. To reduce autofluorescence, slides were then incubated with 1% sodium borohydride in PBS for 2 mins at RT. Slides were then blocked with 10% serum (goat or donkey) in 1× PBS with 0.25% TritonX-100 for 1 h followed by primary antibody diluted in blocking buffer overnight at 4 °C. Slides were then incubated with Dylight conjugated secondary antibodies (1:800; Jackson Immunoresearch) or Alexa flour (1:500; Invitrogen) conjugated secondary antibodies for 1 h at RT in the dark, washed with 1× PBS, incubated with Hoechst staining solution (2 μg/ml in PBS), and then mounted with Aqua Polymount (Polysciences).

Biotin-streptavidin amplification was done by incubating slides with biotinylated antibody (1:500; Vector Labs) for 1 h at RT followed by Dylight conjugated to streptavidin (1:100; Vector Labs) for 1 h at RT, in lieu of Dylight or Alexa flour conjugated secondary antibodies. For co-labeling involving primary antibodies made in the same host, tyramide signal amplification was performed as per manufacturer’s protocol (Perkin Elmer). For primary antibodies made in mouse, reagents from the mouse on mouse kit (Vector Labs) were used for blocking and primary antibody dilution. Labeled slides were imaged using Olympus Fluoview FV 1000. Antibodies used for immunofluorescence are listed in Table 2. Cell counts were performed using the cell counter plugin of ImageJ. 40× images (*n* = 9) of retinal sections labeled with SOX9, CALBINDIN, CHX10, and BRN3A and 60× image of retinal flatmounts (*n* = 8) labeled for GαTRANSDUCIN were used for cell count analysis. Retinal thickness was measured on cross section of retina 200 μm away from the optic nerve.

**Table 2:** List of antibodies

Marker	Company	If concentration	Flatmount concentration	Western blot concentration
IBA1	WAKO	1:500	1:250	
PU.1	CELL SIGNALING	1:100		
SOX9	MILLIPORE	1:500		
GαT1	SANTA CRUZ		1:100	
CALBINDIN	SIGMA	1:250		
CHX10	EXALPHA	1:500		
BRN3A	CHEMICON	1:250		
GFAP	DAKO (polyclonal)	1:250	1:100	
GFAP	DAKO (monoclonal)			1:1000
S100β	ABCAM	1:300		1:1000
NCAN	R&D SYSTEMS	1:100		
TXNIP	SANTA CRUZ			1:250
phospho-SMAD1/5/9	CELL SIGNALING	1:100		
phsopho-TAK	ABCAM	1:500		
β - TUBULIN	SIGMA			1:1000
GFP	THERMO SCIENTIFIC	1:150		

### Western Blotting

Extraction of proteins from retinal tissue was performed using lysis buffer as previously described in [11]. Briefly, retinal tissue was homogenized in PBS and centrifuged at 13,000 rpm, 4 °C for 10 min. The supernatant was discarded, and the pellet incubated with lysis buffer (150 mM NaCl, 50mMTris pH 8.0, 2 mM EDTA, 5% TritonX-100; 100 mM PMSF and protease inhibitor cocktail, RPI corp.) for 20 min at 4 °C. The samples were centrifuged at 13,000 rpm, 4 °C for 10 min, and total protein was estimated using BCA protein assay kit (ThermoScientific).

Forty micrograms of protein was loaded onto 4–20% SDS precast gels (Expedeon), placed in a Biorad gel run apparatus, and run at 150 V for 1 h. Proteins were transferred onto a PVDF membrane, which was blocked with a 5% milk in tris buffered saline tween 20 (TBST) at RT for 1 h on a shaker. The blots were incubated with primary antibody-diluted TBST at 4 °C overnight on a shaker. The following day, the blots were washed in TBST and incubated with HRP conjugated secondary diluted 1:5000 in TBST for 1 h at RT. Blots were washed in TBST, incubated with super signal west femto chemiluminescent substrate (ThermoScientific), and visualized on x-ray films. β-TUBULIN was used as a loading control, and the concentrations of antibodies used are listed in Table 2.

### ELISA

Enzyme linked immunosorbent assay (ELISA) for IFN-γ was performed on media from treated cells in vitro or from whole mouse retina protein lysates using the mouse IFN-γ ELISA kit (Cat # ENEM1001, ThermoScientific) as per manufacturer’s protocol.

### Retinal flatmounts

Preparation of retinal flatmounts and immunolabeling was done as described in [46]. Briefly, enucleated eyes were washed in 1× PBS, fixed in 4% PFA for 15 min, transferred to 2× PBS on ice for 10 min, and followed by retina isolation. Four to five radial incisions were made in the retina to create a petal shape. Excess PBS was absorbed, and retinas were transferred to cold methanol (−20 °C) for 20 min. The tissue was washed with 1× PBS and blocked in Perm/Block solution (1× PBS, 0.3% TritonX-100, 0.2% bovine serum albumin, and 5% donkey or goat serum). The tissue was then washed in PBSTX (1× PBS + 0.3% TritonX-100) and incubated with primary antibody (Table 2) overnight at 4 °C. On the following day, the tissue was washed in PBSTX, incubated with secondary antibody, washed, incubated with Hoechst solution, and mounted onto a slide with Aqua Polymount (Polysciences, Inc). Labeled slides were imaged using Olympus Fluoview FV 1000. Morphological analysis of labeled microglia (*n* = 4 per time point) for changes in area and number of branches was performed using the Scholl analysis plugin in Fiji image analysis software [47]. Briefly, the flatmount image was loaded on to the Fiji software and converted to binary. To calculate the area of the cell, the “Measure” plugin was selected from the “Analyze” options. To determine the number of branches, a center of analysis was defined via the straight line method. This line was drawn from the center of the cell to the end of the longest branch to define a valid “Startup ROI.” The program was run on the default parameters with the starting radius set at 10 pixels.

### Statistical analysis

Statistical analysis was performed via unpaired Students’s *t* test using SPSS software (IBM) between control/vehicle and treated groups for RT-qPCR, cell counts, and microglia morphology. RT-qPCR and densitometries from PLX and control mice injected with vehicle or BMP7 were analyzed via one way ANOVA with Tukey’s test for post hoc analysis. *p* ≤ 0.05 were considered to be statistically significant.

## Results

### BMP signaling in retinal microglia

**Fig. 1:**
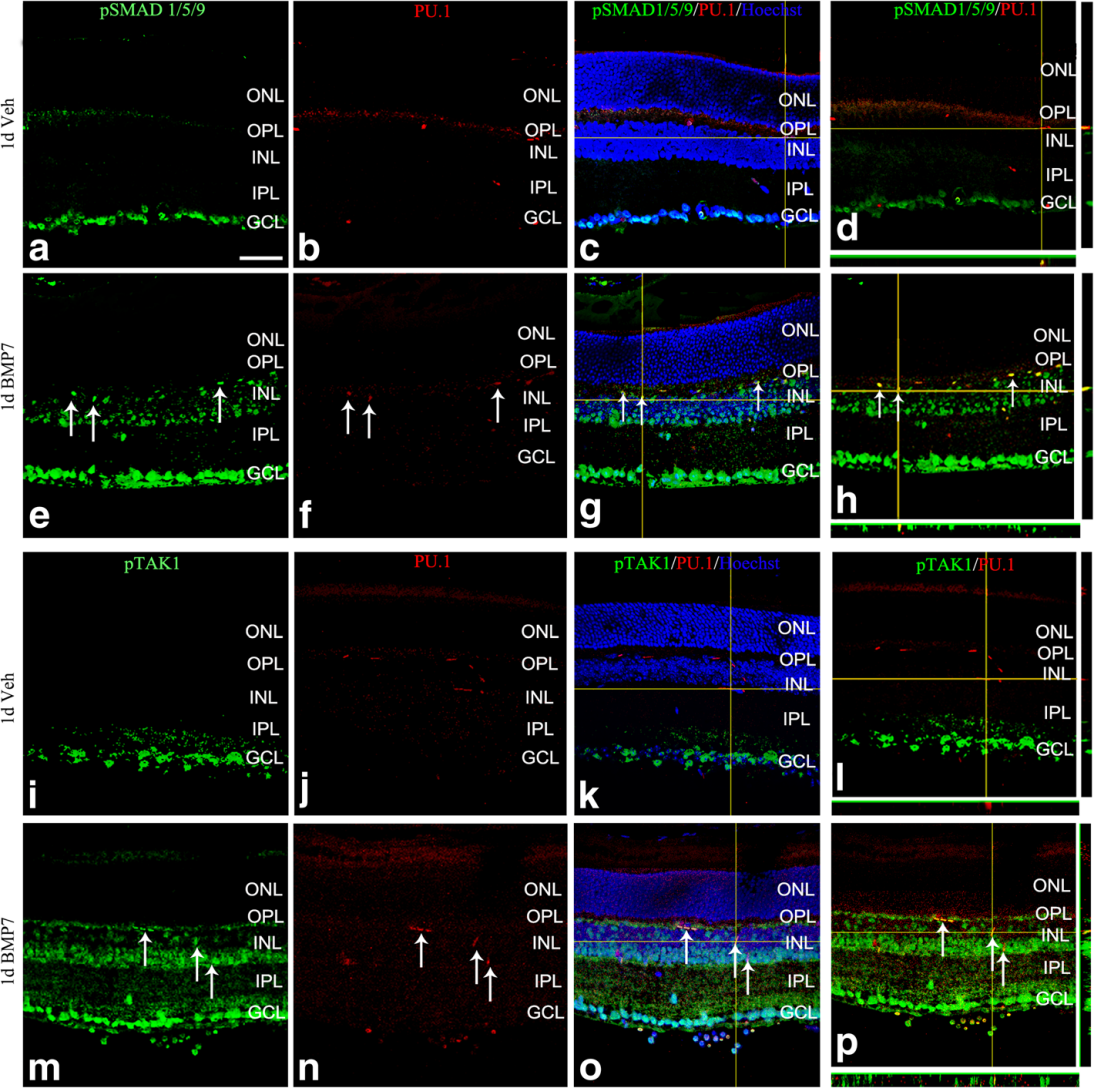
pSMAD and pTAK1 are localized to retinal microglia. Retinal sections from P30 mouse injected with vehicle or BMP7 24 h postinjection were double-labeled with antibodies that label microglial nuclei (PU.1) and phospho SMAD 1/5/9 (pSMAD; **a**–**h**) or phospho TAK1 (pTAK1; **i**–**p**). Thin plane confocal microscopy images with *y*,*z* (*strips* to right of the panel) and *x*,*z* planes (*strips* at the bottom of the panels) shown in (**d**), (**h**), (**l**), and (**p**). pSMAD-labeled cells were primarily found in the GCL in the vehicle-treated retina, with some colocalization with the nuclear microglial marker PU.1 (**a**–**d**). The BMP7-injected retina had an increase in pSMAD expression in the INL as well as substantial colocalization with PU.1 (**e**–**h**). In contrast, vehicle-injected retina showed pTAK1 expression in the GCL with little to no PU.1 colocalization (**i**–**l**), while the BMP7-injected retinas showed increased levels of pTAK1 levels in the INL, as well as significant colocalization with PU.1 (**m**–**p**). *Magnification bar* in **a** = 50 μm, for images (**a**–**p**)

Previous studies have shown that BMP7 triggers reactive gliosis of the retinal macroglia. Both the canonical as well as the non-canonical BMP-MAPK pathways were active in the retinal Müller cells and astrocytes following BMP7 treatment [11]. However, the mechanism by which BMP7 triggered gliosis remains unclear. To determine if any of these pathways were activated in the microglia of control- or BMP7-treated retina, double-label immunohistochemistry was performed using antibodies to phospho SMAD 1/5/9 (pSMAD), phospho TAK1 (pTAK1), and PU.1 (a nuclear marker of microglia; Additional file 1: Figure S1) on adult retinas following intravitreal injection of vehicle or BMP7. In both vehicle- (Fig. 1a–d) and BMP7-treated retinas (Fig. 1e–h), sections showed nuclear co-labeling with PU.1 and pSMAD. In contrast, pTAK1 was localized primarily to the nuclei of GCL of vehicle-injected retinas with no apparent co-localization with PU.1 (Fig. 1i–l), but co-labeled PU.1+ cells in the BMP7-treated retinas, in addition to other cells in the INL and GCL (Fig. 1m–p). There was also a striking increase in the localization of pTAK1 in both the inner and outer plexiform layers of BMP7-treated retinas that was not apparent in vehicle-treated retinas (Fig. 1m–p). Retinal sections were also co-labeled with IBA1 and pTAK1 or pSMAD to show localization in microglia (Additional file 2: Figure S2). Negative controls showed no label (Additional file 3: Figure S3).

### BMP7 induces inflammatory changes in vivo

**Fig. 2:**
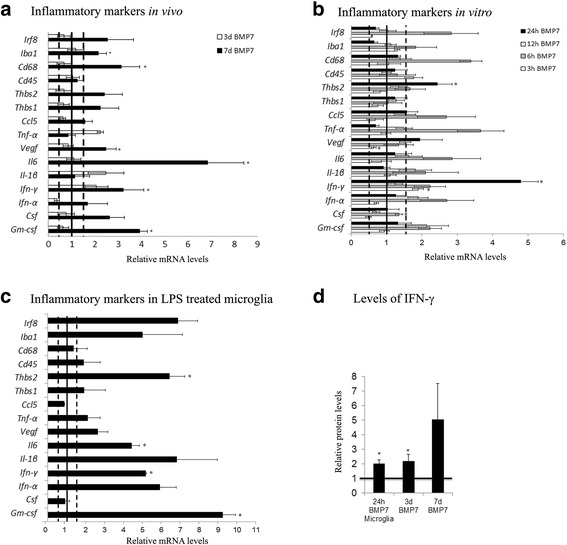
BMP7 injection triggers inflammatory changes in the mouse retina. Expression levels of a panel of proinflammatory markers were analyzed by RT-qPCR in RNA samples from mouse retina injected with vehicle or BMP7, harvested 3 and 7 days postinjection (**a**). At 3 days post-BMP7 injection, about a 2-fold increase in RNA levels, relative to the vehicle controls, was observed in levels of *Ifn-γ*, *Tnf-α*, and *Il-1β*. Seven days post-BMP7 injection, 2-fold increase in levels was observed in *Csf*, *Vegf*, *Thbs1*, and *Thbs2*, and greater than 3-fold increase in *Gm-csf*, *Ifn-γ*, *Il6*, and *CD68* RNA levels relative to the vehicle-injected control. Mouse retinal microglial cells treated with BMP7 for 3, 6, 12, and 24 h were also analyzed for changes in RNA levels of inflammatory markers (**b**), with LPS treatment used as a positive control (**c**). In vitro treatments showed a significant increase in *Ifn-γ* levels at the 3-h time point. At 6 h post-BMP7 treatment, mRNA levels of *Gm-csf*, *Ifn-γ*, *Csf*, *Tnfα*, *Il-6*, and *Cd68* were increased to 1.5-fold or greater. By 12 h, we observed no significant differences between BMP7 and vehicle-treated samples. At the 24-h time point, however, we observed significant increases in the levels of *Ifn-γ* and *Thbs*. The LPS-treated microglia showed a relative increase in most of the markers, with significant increases observed in levels of *Gm-csf*, *Ifn-γ*, *Il-6*, and *Thbs2* (**c**). Protein levels of IFN-γ was also determined via ELISA (**d**). We observed a 2-fold increase in levels in medium from microglial cells incubated with BMP7 for 24 h and in protein from whole retinal tissue from mice injected with BMP7 for 3 days, when compared to their respective vehicle control. Protein from 7 days BMP7-injected retina showed a 5-fold increase in protein levels compared to the vehicle control. Data shown in graphs represent relative expression levels of RNA or protein of BMP7 or LPS-treated samples to their respective vehicle control. Bars above a level of 1.0 (*solid black line*) represent an increase in mRNA levels while bars below the level of 1.0 represent a decrease in mRNA levels relative to the corresponding vehicle control. Statistical analysis was performed by unpaired Student’s *t* test. *p value <0.05. Abbreviations: *CD* cluster of differentiation, *Csf* colony stimulating factor, *Gm-csf* granulocyte macrophage colony stimulating factor, *Ifn* interferon, *Il* interleukin, *Tnf-α* tumor necrosis factor alpha, *Thbs* thrombospondin, *Vegf* vascular endothelial growth factor

To determine whether BMP7 regulated inflammatory signals that could then either trigger or enhance the gliosis response, BMP7-treated retinas were analyzed for messenger RNA (mRNA) levels of proinflammatory markers (Fig. 2a). For the analyses of mRNA levels, values plotted in graphs were all relative to control levels which were set to a value of 1.0; hence, increases in mRNA levels in comparison to controls are bars above a level of 1.0, while a decrease is represented by bars below the level of 1.0. Three days postinjection, increases of 1.5-fold or more in mRNA levels of *Tnf-α*, *Il-1β*, and *Ifn-γ* were present. However, larger increases were evident in multiple factors 7 days postinjection, including granulocyte macrophage colony stimulating factor (*Gm-Csf)*, colony stimulating factor (*Csf*), *Ifn-α*, *Ifn-γ*, *Il-6*, *Vegf*, thrombospondins-1 and-2 (*Thbs1 & Thbs2*), and *Cd68*. We also observed more than a 2-fold increase in microglial marker *Iba1* and *Irf8*, markers for activated microglia.

To determine if the increases in proinflammatory markers present in BMP7-treated retinas were mediated by retinal microglial cells, the effect of BMP7 treatment on isolated mouse retinal microglial cells in vitro was observed using RT-qPCR. mRNA levels were investigated in microglial cells incubated with vehicle or BMP7 for 3, 6, 12, or 24 h (Fig. 2b). Again, changes in mRNA levels relative to controls were plotted, where a value of 1.0 indicates levels of control mRNA. Following 3 h of incubation with BMP7, only levels of *Ifn-γ* were 1.5-fold greater, whereas at 6 h the average mRNA levels of *Gm-csf*, *Ifn-γ*, *Csf1*, *Tnf-α* and *Il-6*, and *Cd68* were increased to 1.5-fold above control or greater (Fig. 2b). By 24 h of incubation, many of the molecule levels were decreased in comparison to the 6-h time point; however, *Ifn-γ* and *Thbs2* were increased in comparison to control and 6-h mRNA levels. As a positive control for inflammation, microglia were incubated with LPS for 3 h (Fig. 2c). To determine if the changes in RNA levels are being translated to protein, we determined IFN-γ levels by an ELISA using medium from microglial cells incubated with BMP7 for 24 h and whole retinal lysates from mice treated with vehicle or BMP7 (Fig. 2d). Values plotted in graph are relative to the respective vehicle controls; hence, increases in mRNA levels in comparison to controls are bars above a level of 1.0, while bars below the level of 1.0 represent a decrease. We observed a 2-fold increase in the IFN-γ protein levels in the astrocytes and microglial cell medium, and a 5-fold increase in IFN-γ protein level was detected in retinal lysates 7 days posttreatment with BMP7 compared with vehicle.

**Fig. 3:**
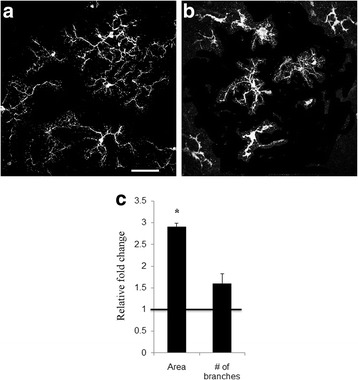
BMP7 alters microglial morphology. Retinal flatmounts from 1 day BMP7- and vehicle-injected retina were labeled for IBA1 (**a**, **b**) and analyzed for morphological changes. An increase in the area of the microglia was observed when compared to the vehicle control (**c**). Number of branches and branch length were also assessed for the treated cells, and increase in the number of branches was observed with a decrease in the branch length of the cells incubated with LPS or BMP7 when compared to the vehicle control (**c**). Data shown in **c** represent relative change in area and number of branches in BMP7-treated samples to the vehicle control. Bars above a level of 1.0 (*solid black line*) represent an increase while bars below the level of 1.0 represent a decrease in the parameter measured, relative to the corresponding vehicle control. *Magnification bar* in **a** = 50 μm, for images (**a**) and (**b**). Abbreviation: *IBA1* ionized calcium-binding adapter molecule 1

Changes in morphological characteristics of microglia following control and BMP7 treatments were subsequently investigated. It has been reported by other investigators that activated microglia increase in area with an increase in branch points [48]. Retinal flatmounts of 1 day BMP7- and vehicle-treated retinas were labeled with IBA1 and analyzed for average cell area and number of branch points in cellular processes (Fig. 3a, b). Graphs show relative changes in the area and number of branches (“Median intersections” output from the Sholl analysis). Morphological analysis revealed that the BMP7-treated retinas contained microglia with a larger area in comparison to vehicle-treated retinas, and a decrease in the number of branches (Fig. 3c).

### Activated microglia secrete factors that induce gliosis

**Fig. 4:**
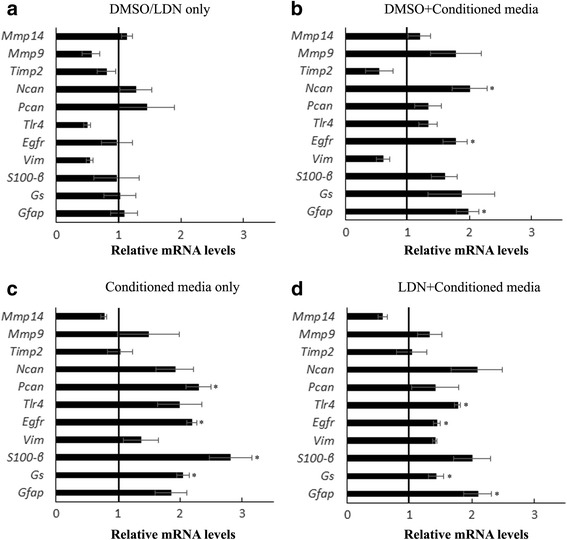
Activated microglia secrete factors that trigger retinal gliosis. Conditioned medium from microglial cells incubated with BMP7 or vehicle for 24 h was added to the medium of the retinal astrocytes, directly or pretreated with LDN193189 (**c**, **d**). RNA was isolated from these cells 24 h posttreatment and analyzed via RT-qPCR for a panel of gliosis markers. Statistically significant increase in levels of *Gfap*, *Gs*, *S100-β*, *Pcan*, *Egfr*, and *Tlr4* was observed in astrocytes incubated with conditioned medium added directly or pretreated with LDN193189 (**c**, **d**). Cells treated with DMSO (carrier for LDN193189) or LDN only (**a**) or cells pretreated with DMSO and conditioned medium from BMP7 or vehicle-treated microglia (**b**) were used as experimental controls. Data shown in graphs represent relative expression levels of RNA in retinal astrocyte cells treated with LDN193189 relative to DMSO (**a**) or with conditioned media from BMP7-treated microglial cells relative to retinal astrocyte cells treated with conditioned media from vehicle-treated microglia (**b**–**d**). Bars above a level of 1.0 (*solid black line*) represent an increase in mRNA levels while bars below the level of 1.0 represent a decrease in mRNA levels relative to the corresponding vehicle control. Statistical analysis was performed by unpaired Student’s *t* test. *p value <0.05. Abbreviations: *Egfr* epidermal growth factor receptor, *Gfap* glial fibrillary acidic protein, *Pcan* phosphacan, *Tlr* toll like receptor

We have observed that BMP7 is able to activate retinal microglia in vitro and in vivo (Figs. 2 and 3, respectively). To determine if microglia secrete factors that trigger reactive gliosis in vitro, we used conditioned medium obtained from mouse microglia cultures treated with vehicle (vehicle conditioned media) or BMP7 (BMP7-conditioned media) for 24 h, and used for treatment of mouse retinal astrocytes (Fig. 4b–d). Graphs represent mRNA levels in astrocyte cultures treated with BMP7-conditioned media relative to cultures treated with vehicle-conditioned media; pretreated with DMSO or LDN193189. Retinal astrocyte cells were incubated for 24 h with microglial cell-conditioned medium and were assessed for changes in markers associated with gliosis. To reduce the possibility that the BMP7 added to the microglial medium might directly affect the astrocytes, an inhibitor of BMP receptors, LDN193189, was added to the conditioned medium (Fig. 4d). RT-qPCR analysis showed a statistically significant increase in expression of gliosis markers glial fibrillary acidic protein (*Gfap*), *S100-β*, *Gs*, epidermal growth factor receptor (*Egfr*), and phosphacan (*Pcan*) 1.5-fold above that of astrocyte cells treated with DMSO and vehicle-treated conditioned media (Fig. 4b). When BMP inhibitor was added to the astrocyte medium prior to addition of conditioned medium from microglia, statistically significant increases were detected in *Gfap*, *Gs*, *S100-β*, *Egfr*, and toll like receptor-4 (*Tlr4*; Fig. 4d). Treatment of retinal astrocytes with DMSO or LDN alone or with conditioned media in presence of DMSO were used as experimental controls (Fig. 4a, c). We did not observe any changes when cells were treated with LDN alone (Fig. 4a). Treatment of retinal astrocytes with conditioned media in the presence of DMSO showed similar changes in expression as cells treated with conditioned media alone (Fig. 4c).

### PLX ablates retinal microglia

**Fig. 5:**
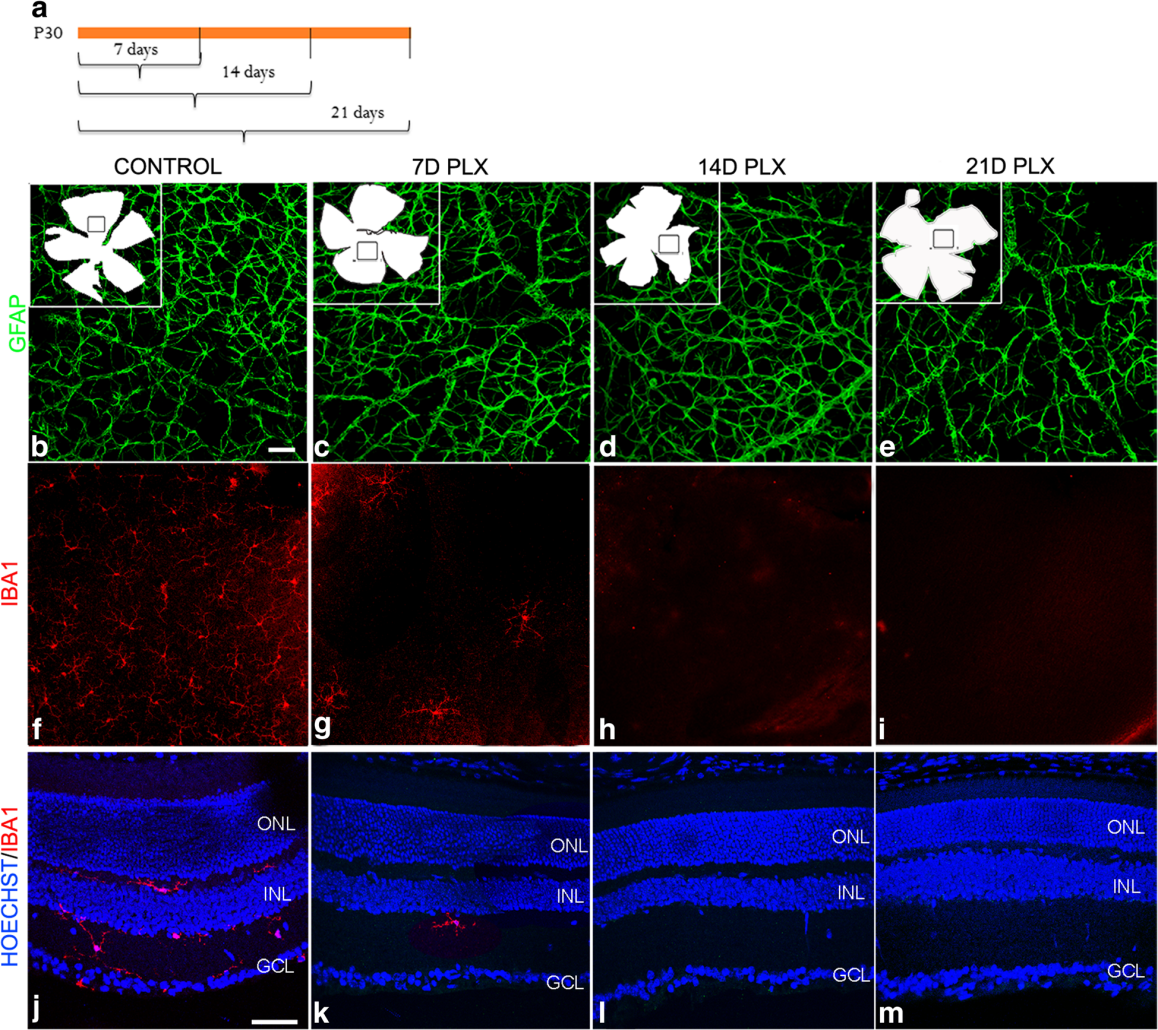
PLX ablates microglia in the retina. Mice were fed with chow-containing PLX or vehicle dye to determine ablation of microglia in the retina. **a** Schematic describing the time points for which the mice were fed with chow-containing PLX, following which eyes were harvested. Retinal flatmounts prepared from the eyes harvested at 7, 14, and 21 days were labeled for GFAP or IBA1 (**b**–**i**). *Insets* in **b**–**e** indicate the flatmount outline and from where the images (**b**–**i**) was taken. While GFAP did not show any difference between the stages examine (**b**–**e**), there was a significant decrease in IBA1 label in mice kept on PLX diet for 7 days (**f**, **g**). By 14 days, no IBA1 label was found in the retinal flatmount and this absence persisted into the 21-day time point (**h**, **i**). Retinal sections control and PLX-treated mice labeled for IBA1 to show loss of microglia in the deeper layers of the retina (**j**–**m**). Statistical analysis was performed by unpaired Student’s *t* test. *p value <0.05. *Magnification bar* in **b** = 50 μm, for images **b**–**i**. *Magnification bar* in **j** = 50 μm, for images **j**–**m**. Abbreviations: *GFAP* glial fibrillary acidic protein, *IBA1* ionized calcium-binding adapter molecule 1

**Fig. 6:**
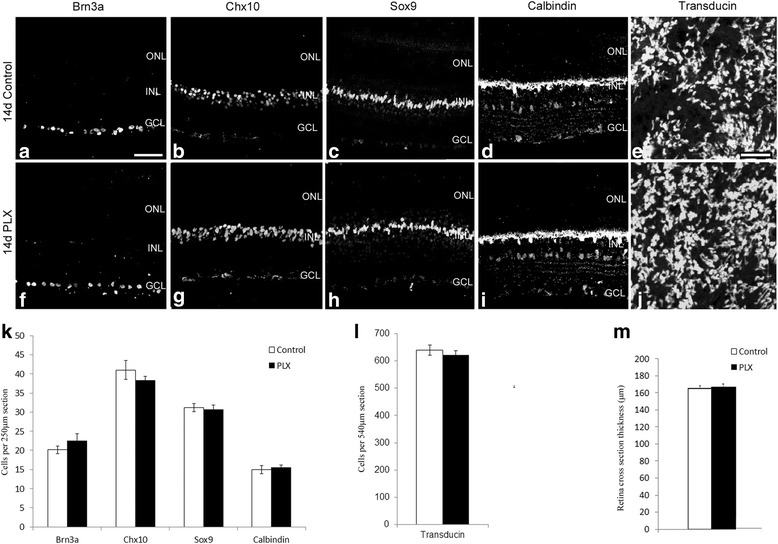
PLX ablates microglia without affecting other retinal cells. Retinal sections from mice kept on the PLX or vehicle chow diet for 14 days were labeled for ganglion cells (Brn3a; **a**, **f**), bipolar cells (Chx10; **b**, **g**), Müller glia (Sox9; **c**, **h**), and horizontal cells (Calbindin; **d**, **i**). Cell counts of images for the labeled markers (*n* = 8) showed no difference between the control and PLX-treated mice (**k**; Images taken were within 200 μm from the optic nerve). 60× images of retinal flatmounts of PLX and control-treated mice were labeled for Gα transducin (**e**, **j**). Cell counts showed no difference in the two treatments (**l**). Retinal thickness was also assessed in control and PLX retinas and showed no difference (**m**). *Magnification bar* in **a** = 50 μm, for images (**a**–**d**, **f**–**i**). *Magnification bar* in **e** = 10 μm, for images (**e**, **j**)

To further investigate the role of microglia in BMP7-mediated gliosis, a means to ablate microglial cells within the retina was sought. Previous reports have shown colony stimulating factor receptor 1 (CSFR1) inhibitor, PLX3397, to selectively ablate microglia in the brain [49]. We have used a variant of the drug, PLX5622, supplied by Plexxikon Inc. in chow form to determine its effect on retinal microglia. Starting at postnatal day 30, mice were switched to control chow or chow containing 1200 ppm PLX. The mice continued treatment with the inhibitor-laced chow until sacrificed 7, 14, or 21 days later. Retinal flatmounts from control and PLX mice were isolated for 7, 14, and 21 days, and labeled for IBA1 and GFAP (Fig. 5). Although no apparent change in GFAP was observed (Fig. 5b–e), there was a clear decrease in the number of IBA1+ cells 7 days after starting the PLX diet, and IBA1 immunoreactivity was completely lost by 14 days (Fig. 5f–m). Retinal tissue sections from these mice were also analyzed for ganglion cells (Brn3a), bipolar cells (Chx10), Müller glia (SOX9), and horizontal cells (Calbindin) (Fig. 6a–d, f–i). Cell counts for labeled cells showed no statistically significant change between the control and PLX-treated mice (Fig. 6k). Retinal flatmounts of PLX and vehicle-treated retinas were also labeled with Gα transducin to label photoreceptors (Fig. 6e, j). Cell count of labeled images showed no statistically significant change in cell numbers (Fig. 6l). Thickness of retinal sections of the control and PLX treated mice also showed no change (Fig. 6m).

### Microglial ablation reduces BMP7-mediated gliosis

**Fig. 7:**
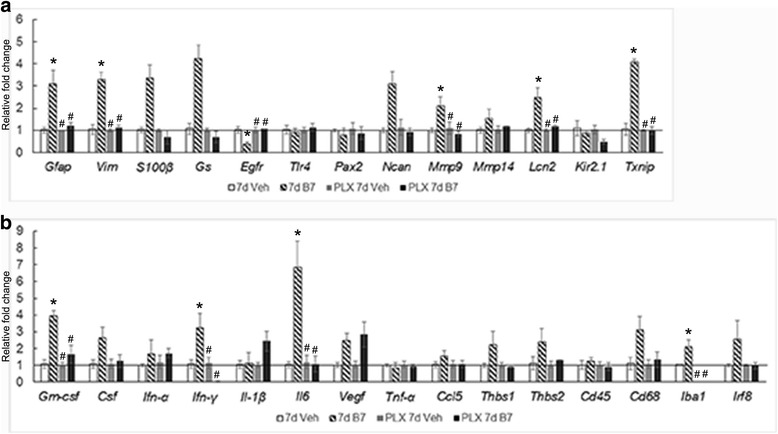
Effect of BMP7 is diminished in the absence of microglia—RNA levels. BMP7 or vehicle was injected intravitreally into the eyes of mice kept on regular chow or PLX chow and harvested 7 days postinjection. RNA isolated from the retina were analyzed via RT-qPCR for changes in levels of inflammatory markers (**a**) and gliosis markers (**b**). Mice kept on the control chow and injected with BMP7 showed a relative increase by 2-fold or greater of inflammatory markers: *Gm-csf*, *Csf*, *Ifn-γ*, *Il-6*, *Vegf*, *Thbs1*, *Thbs2*, and *CD68* (**b**). Gliosis markers *Gfap*, *Vim*, *S100-β*, *Gs*, *Ncan*, *Mmp9*, *Lcn2*, and *Txnip* showed a 2-fold increase or more in these mice (**a**). Data shown in graphs (**a**, **b**) represent relative expression levels of RNA in mouse retina to the respective vehicle treatments. Bars above a level of 1.0 (*solid black line*) represent an increase in mRNA levels while bars below the level of 1.0 represent a decrease in mRNA levels relative to the corresponding vehicle control. Mice kept on the PLX chow and injected with BMP7 showed a 2-fold increase in inflammatory markers *Il-1β* and *Vegf*, while all the gliosis markers showed relatively unchanged RNA levels (**a**, **b**). Statistical analysis was performed by one way ANOVA with post hoc Tukey test. Significant difference from vehicle-injected control mice *p value <0.05. Significant difference from BMP7 injected control mice #*p value* <0.05. Abbreviations: *Csf* colony stimulating factor, *Egfr* epidermal growth factor receptor, *Gm-csf* granulocyte macrophage colony stimulating factor, *Gfap* glial fibrillary acidic protein, *Gs* glutamine synthetase, *Ifn-γ* interferon-gamma, *Il* interleukin, *Lcn* lipocalin, *Mmp* matrix metalloproteinase, *Ncan* neurocan, *Pcan* phosphacan, *Txnip* thioredoxin interacting protein, *Tnf-α* tumor necrosis factor alpha, *Thbs* thrombospondin, *Tlr* toll like receptor, *Vim* vimentin, *Vegf* vascular endothelial growth factor

**Fig. 8:**
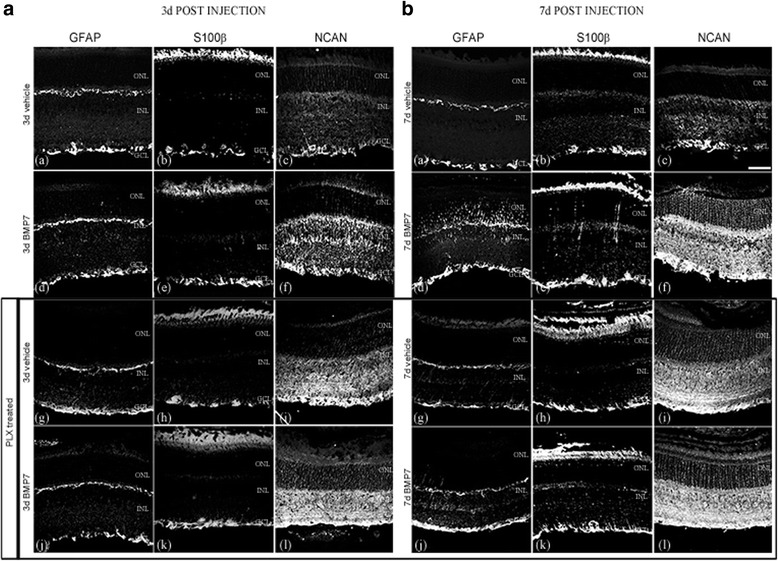
Effect of BMP7 on gliosis in absence of microglia—localization of gliosis markers. Mouse retinal sections from eyes injected with vehicle or BMP7 were labeled for gliosis markers GFAP (**A**, **B** (**a**, **d**, **g**, **j**)), S100-β (**A**, **B** (**b**, **e**, **h**, **k**)), and NCAN (**A**, **B** (**c**, **f**, **j**, **l**)). Mice kept on the PLX diet did not show an increase in label for the gliosis markers GFAP and S100-β BMP7 or vehicle-injected retina 3 and 7 days postinjection (**A**, **B** (**g**, **h**, **j**, **k**)). NCAN label appeared to be similar in the BMP7 injected and the respective age-matched vehicle controls in mice kept on the PLX chow (**A**, **B** (**i**, **l**)). Mice kept on the control chow and injected with BMP7 clearly showed an increase in GFAP, S100-β, and NCAN levels 7 days postinjection, when compared to their respective vehicle control (**B** (**a**–**f**)). Three days postinjection, there is an increase in GFAP and NCAN label in BMP7-injected retinas in comparison to the respective vehicle control-injected retinas (**A** (**a**, **c**, **d**, **f**)). When comparing mice kept on control chow or the PLX chow, there is an increase in GFAP and S100-β label in the mice kept on control chow in comparison to the mice kept on the PLX chow, 7 days post BMP7 injection (**B** (**d**, **e**, **j**, **k**)). GFAP and S100-β label in mice kept on control chow and PLX chow appears to be similar in the BMP7-injected retinas, 3 days postinjection (**A** (**d**, **e**, **j**, **k**)). *Magnification bar* in (**B**, (**c**)) = 50 μm, for images **A**, **B** (**a**–**l**)

To determine if microglia were involved in BMP7-mediated gliosis response, mice with ablated microglia (PLX mice) were injected intravitreally with vehicle or BMP7, and mRNA levels of proinflammatory markers or gliosis-related molecules were determined by RT-qPCR 7 days postinjection. As in previous graphs, levels of mRNA are relative to levels in the respective vehicle-treated mice. Mice kept on control chow and treated with BMP7 showed an increase in levels of inflammatory markers including *Gm-csf*, *Ifn-γ*, *Il-6*, and *Iba1* and gliosis markers including vimentin (*Vim*), *Gfap*, *Egfr*, *Mmp9*, lipocalin 2 (*Lcn2*), and thioredoxin interacting protein (*Txnip)* (Fig. 7a, b). Analysis of inflammatory markers of mice on PLX chow and treated with BMP7 via RT-qPCR also showed only modest increases in levels of *Il-1β* and *Vegf* compared to vehicle control (Fig. 7b). mRNA levels of *Gm-csf*, *Ifn-γ*, *Il-6*, *Cd68*, and *Iba1* dropped drastically when microglia were not present (Fig. 7b). RT-qPCR analysis of PLX mice 7 days post-BMP7 treatment showed no increase in mRNA levels of gliosis markers compared to the PLX vehicle controls (Fig. 7a). Markers indicative of gliosis were further investigated by examining patterns of immunoreactivity for GFAP, S100-β, and neurocan (NCAN) (Fig. 8A, B). Three days following vehicle or BMP7 injection, the PLX mice showed similar levels of GFAP and S100-β label in control and PLX mice (Fig. 8A (a, b, d, e, g, h, j, k)). However, 7 days postinjection, the PLX mice showed decreased GFAP and S100-β label in BMP7-injected PLX retinas (Fig. 8B (j, k)), when compared to the control BMP7-injected retinas (Fig. 8B (d, e)). NCAN immunofluorescence label did not diminish following PLX treatment in comparison to controls at either 3 days (Fig. 8A (f, l)) or 7 days postinjection (Fig. 8B (f, l)). Moreover, levels of NCAN were increased in vehicle-injected eyes at both 3 and 7 days of PLX-treated mice in comparison to vehicle-injected eyes of control-treated mice (compare Fig. 8A (c and i) and Fig. 8B (c and i)), supporting a potential role for microglia in extracellular matrix remodeling. Negative controls showed no label (Additional file 3: Figure S3). Gliosis markers showed similar label in uninjected mice in comparison to the 3 day and 7 day vehicle injected mice (Additional file 4; Figure S4). Protein levels of gliosis markers GFAP, S100-β, and TXNIP were also quantified using western blot (Additional file 5: Figure S5).

## Discussion

Our lab previously showed that BMP7 is able to trigger reactive gliosis in the retina. Here, we show that the Müller cell gliosis triggered by BMP7 is an indirect effect resulting from microglial activation to a proinflammatory state. Following exposure to BMP7, microglia upregulated at least two molecules, IFN-γ and IL-6, both of which have been shown in previous studies to trigger gliosis [50–54]. The CSFR1 inhibitor PLX was used to specifically target and ablate retinal microglia without affecting numbers of other retinal cells, in order to show that the BMP7 triggers gliosis through microglial activation. We observed that BMP7 injection into retinas lacking microglia produced an abated inflammatory response and a complete loss of gliosis, suggesting an important role for the microglia in mediating the gliosis response.

### BMP pathway in retinal disease

BMPs have been previously shown to be regulated in injury and disease models of the CNS and retina [21, 55–57]. The BMP receptors type 1A and 1B regulate hypertrophic and scarring responses of astrocytes following spinal cord injury [12]. In the retina, BMP signaling components, phospho SMAD 1/5/8, have also shown to be upregulated following NMDA-induced injury and promote retinal ganglion cell’s survival [8]. We observed an increase in pTAK1 label in IBA-labeled cells in the retina, as well as in other cells of the inner nuclear layer. Increases in expression of pTAK1 in neurons have been previously reported in the brain following cerebral ischemia and is known to be expressed in axonal arbors of sensory neurons [58, 59]. BMPs have also been shown to be important in retinal cell proliferation and regeneration in the chick retina [60]. Ueki and Reh [61] showed that SMAD upregulation was essential in mediating EGF dependent Müller glial cell proliferation in the mouse. The presence of BMPs in disease states is consistent with a potential role for them playing a role in retinal gliosis.

### Activated microglia drive retinal gliosis

We had previously reported that BMP7 was able to trigger gliosis in retinal glia in vitro and in vivo. However, we observed a higher response in the in vivo model, which suggested there may be other cells involved in this response. Microglia are the resident macrophages in the retina. Similar to the macroglia, these cells also undergo activation. Their activation has been observed in various disease and injury models, such as retinitis pigmentosa, diabetic retinopathy, retinal detachment, and glaucoma [62–66]. Activated microglia change morphology from a ramified cell to an amoeboid cell, along with changes in expression of cell surface markers, such as the cluster of differentiation molecule 11b (CD11b), CD68, major histocompatibility complexes (MHC), scavenger receptors, and TLR and secreted actors such as RANTES, interferon, interleukins, and TNFα. These changes serve to enhance the phagocytic effect of the microglial cells as well as the cytotoxic effect on injured cells and foreign pathogen [23, 67]. Müller glia also undergo activation following disruption of retinal homeostasis. The reactive Müller glia hypertrophy and upregulated expression of various growth factors, reactive oxygen species scavengers, protect neurons from excitotoxicity and, in some organisms, can regenerate retinal neurons. These changes serve to protect the damaged retina. However, gliosis can also have detrimental effects by remodeling the extra cellular matrix and due to loss of normal glial functions which are necessary for normal neuronal activity [6, 7].

The retinal astrocytes and Müller glial cells exhibit similar responses to injury, such as hypertrophy, upregulation of GFAP, vimentin, and GS, as observed in rat models of retinal detachment and retinitis pigmentosa [68–70]. However, research has also revealed that there are differences in the response of the two cell types. GFAP upregulation was observed in Müller glia and not in the astrocytes in rats subjected to episcleral vein cauterization [71]. Similarly, upregulation of GFAP was observed in the Müller glial cells in the retina subjected to laser-induced ocular hypertension, while the astrocytes of the contralateral control eyes also exhibited an increase in GFAP and a change in the area covered by the astrocytes [72, 73]. The differences observed may suggest distinct functional roles for the astrocytes and Müller glia, which cooperate to restore retinal homeostasis.

Here, we observed a decreased gliosis response in the retina following BMP7 treatment in mice lacking microglia. We used a novel CSF1R inhibitor (PLX) to selectively ablate microglia. Following microglial ablation, mice were treated with BMP7 to assess gliosis in the retina. The inclusion of the inhibitor in the chow allowed its continual application over a longer period of time, enabling the maintenance of a microglia-free environment in the retina in which we could test the role of the microglia in BMP7-mediated gliosis. Without continual application of the inhibitor, microglia could repopulate the retina from one of two sources; bone marrow-derived stem cells can penetrate the blood-brain barrier and differentiate into microglia or residential microglia can proliferate and replace lost cells [74, 75]. The two sources of microglia are not equivalent; residential microglia primarily give rise to microglia that display an M1 inflammatory phenotype, whereas the bone marrow-derived cells give rise to microglia with an M2 anti-inflammatory phenotype [75]. At any rate, in order for us to test the role of BMP7 in indirectly triggering gliosis, we had to maintain a microglial-free environment for the duration of the experiments.

### BMP and inflammation

Activation of microglia and macroglia have been studied in various models. While there are differences in the responses of the two glial populations, they do exhibit similarities. These include regulation of inflammatory markers, antigen presentation complexes, and various factors such as IFN, TNFα, and TLR [3, 6, 36]. While several different factors have been shown to regulate macrophage and microglia activation, the effect of BMPs is still not completely characterized [39, 40, 76, 77]. BMP6 regulates expression of inflammatory markers such as IL-6, IL-1β, and nitric oxide synthase in macrophages [78–80]. In addition, more recent studies indicate that BMP exposure particularly leads to the M2 or anti-inflammatory phenotype of the macrophages promoting tissue repair [81–84]. Microglia are descendants of immature macrophages and are thought to act as macrophages in disease and injury states [85]. In our studies, BMP7 increased the proinflammatory state of the microglia. Further studies are necessary to determine if all microglia respond to BMP7 by increasing proinflammatory markers or if this is a response unique to certain populations of microglia.

In this study, we observed that microglia showed an upregulation of inflammatory markers in response to BMP7 treatment, indicative of activation. Furthermore, in the PLX treated mice, the gliosis response was subdued in comparison to control BMP7 treated retinas, suggesting that microglia are an essential mediator of retinal gliosis. These results support our hypothesis that microglia are activated by BMP7, which in turn regulate factors causing Müller cell gliosis.

In the PLX-treated mice (both vehicle and BMP7-injected), we also observe an increase in neurocan levels in the retina. Müller glia secrete MMPs that regulate neurocan levels in the extracellular matrix. In addition, microglia also secrete these enzymes [86, 87]. Their upregulation has been observed in the CNS during inflammation in various injury models. Furthermore, microglia-derived factors such as TNF-α have also shown to regulate MMP expression by the Müller glia [88]. Thus, we propose that the lack of microglia in the retina contributes to the increase in neurocan by regulating MMP levels either directly or indirectly by regulating Müller glia.

Comparing the mRNA and protein levels in the control and PLX-injected retinas, we observed a difference in expression patterns (Fig. 7, Additional file 5: Figure S5). Although the mRNA levels of S100 and TXNIP was reduced in the BMP7-injected PLX mice, we did not observe a similar change at the protein level. Non-correlation between mRNA and protein levels has been noted in other studies [89–92]. mRNA translation and protein stability in the cell is regulated by multiple systems including micro RNAs (miRNAs), mRNA localization translational repression, and protein stability [92, 93]. miRNAs have been previously reported to be regulated in neural tissue under conditions of stress [94–96]. Furthermore, BMPs can regulate translation by regulating cytoplasmic polyadenylation element binding protein (CPEB) via the TAK pathway [97, 98]. Further studies will be required to determine what pathway(s) mediate this non-correlation between the mRNA and protein levels.

### Microglia release inflammatory factors prior to formation of gliosis

We observed a decrease in expression of GFAP and S100-β in mice kept on the PLX diet and treated with BMP7. BMP7 treatment also revealed decreased RNA levels of gliosis and inflammatory markers in PLX mice when compared to the mice kept on the normal diet. Previously, it has been reported that microglia respond early to changes in microenvironment and become activated. Bosco et al. showed that microglia become activated early in the retina, prior to any increases in IOP in the DBA/2J mice [25]. Similarly, early activation of microglia has also been observed and implicated in progression of Parkinson’s disease [99]. Furthermore, in the ocular hypertension mouse model studied in Gallego et al., the authors suggest that upregulation of MHC-II in microglia in the controlateral eye regulated the morphological changes of retinal astrocytes [73]. Thus, we propose that microglia respond to the BMP7 first and become activated. These activated microglia upregulate factors, which in turn can trigger Müller cell gliosis. Consistent with this notion, our findings indicate the *Ifn-γ* and other inflammatory factors were upregulated as early as 3 h following incubation of microglial cells with BMP7 in vitro, and these levels were further increased 6, 12, and 24 h postincubation with BMP7. In contrast, factors associated with gliosis do not begin to increase until 3 days in vivo, with most markers increasing after 7 days.

### Potential factors regulating microglia-mediated activation of Müller glia

Previous studies looking into microglia and macroglia interactions have revealed several secreted as well as membrane bound factors which could activate the macroglia, such as IL-1β, IL-18, TGF-β, and TNF-α [23, 100]. Morphological changes and increases in RNA levels of inflammatory markers in the microglia following BMP7 treatment indicate activation of the microglia. We observed in our analysis that RNA levels of *Ifn-γ*, *Il-6*, *Vegf*, and *Thbs1* to be greater following Müller glia activation. Previously, Cotinet et.al and Goureau showed that IFN-γ can trigger Müller glia to regulate TNF-α and nitric oxide (NO) [101, 102]. Similarly, IL-6 has been shown to induce Müller glia-derived progenitor cells in the injured zebrafish and chick retina [40, 103]. We propose that BMP7 causes activation of microglia, which leads to upregulation of factors such as IFN-γ and IL-6, which in turn trigger Müller cell gliosis.

Our findings indicated an important role for microglia in Müller cell gliosis in the murine retina. However, the mechanism and potential factors that play a role in microglia and Müller glia interactions are not known. Future studies will aim to identify the potential role of IFN-γ and IL-6, upregulated by BMP7 in the retina, in microglia function, and gliosis.

## Conclusions

Our findings indicate that retinal microglia are essential in regulating retinal gliosis. The expression of downstream BMP signaling components in the retinal microglia, as well as the decrease in retinal gliosis in PLX5622-treated mice demonstrate that BMP7 can regulate gliosis indirectly by activating the retinal microglia. Additionally, we show that regulation of retinal gliosis by microglia could be mediated by IFN-γ or IL-6. Further studies will help evaluate the role of these factors in this response.

## Additional files

Additional file 1: Figure S1. PU.1 localizes with retinal microglia. Co-label of PU.1 antibody with antibody against GFP that cross-reacts with YFP in a retinal section from P30 mice which have YFP tag on vascular endothelial cadherin (VE-YFP), a marker expressed in endothelial cells (A-D). No co-label of PU.1 was observed with VE-YFP. PU.1 was also co-labeled with microglia marker IBA1 to show localization was restricted to microglial cells (E-H). Hoechst merged with the images of green and red channels are shown in D and H. Magnification bar in E = 50 μm, for images A–H. (TIF 857 kb)

Additional file 2: Figure S2. Expression of BMP signaling molecules in microglia in vehicle and BMP7-injected retinas. Retinal sections from P30 mouse injected with vehicle or BMP7 24 h postinjection were double-labeled with antibodies that labels microglia cytoplasm (IBA1) and phospho SMAD 1/5/9 (pSMAD; A–F) or phospho TAK1 (pTAK1; G–L). Thin plane confocal microscopy images with y,z (strips to right of the panel) and x,z planes (strips at the bottom of the panels) shown in C, F, I and L. pSMAD-labeled cells were primarily found in the GCL in the vehicle-treated retina, with some co-localization with the cytoplasmic microglial marker IBA1 (A-C). The BMP7-injected retina had an increase in pSMAD expression in the INL as well as substantial co-localization with IBA1 (D–F). Vehicle-injected retina showed pTAK1 expression in the GCL with little to no IBA1 co-localization (G–I), while the BMP7-injected retinas showed increased levels of pTAK1 levels in the INL, as well as significant co-localization with IBA1 (J–L). Magnification bar in A = 50 μm, for images A–L. (TIF 688 kb)

Additional file 3: Figure S3. Negative control of immunofluorescence labels. Retinal sections from P30 mouse labeled with rabbit immunoglobulin G (Rbt IgG; A–C, D, F), mouse IgG (Mse IgG; E, F), and sheep IgG (G, H) to determine background fluorescence. Images of sections labeled with the nuclear stain, Hoechst merged with the images of green and red channels are shown in C and F. Panels A–C represent sections, which were labeled with IgG following the procedure used for tyramide amplification when using two antibodies for the same species. Images in D–F represent sections co-labeled with rabbit and mouse IgG. Images A–C are negative controls for Fig. 1 and Additional file 1: Figure S1. Images D–F are negative controls for sections labeled with GFAP, S100-β, Calbindin, Brn3a, Chx10, Sox9, and IBA1. Images G and H are negative control sections for NCAN-labeled slides. Magnification bar in A = 50 μm, for images A–H. (TIF 465 kb)

Additional file 4: Figure S4. IF label of retinas for GFAP, S-100-β, and NCAN in P30 uninjected and 3 and 7 days vehicle-injected retinas. Retinal sections from uninjected P30 mouse, vehicle-injected P30 mouse, obtained 3 and 7 days postinjection, labeled for GFAP (A, D, G), S100-β (B, E, H), and NCAN (C, F, I). Label for all three markers appears to be similar in the uninjected and the vehicle-injected retinas. Magnification bar in A = 50 μm, for images A–I. (TIF 5611 kb)

Additional file 5: Figure S5. Protein levels in PLX-treated mice. Protein isolated from control and PLX-treated mice injected with vehicle or BMP7 changes in protein levels of gliosis markers GFAP, S100-β, and TXNIP, with β-Tubulin used as a loading control. GFAP showed elevated levels in the BMP7-injected control mice, while PLX mice had GFAP levels similar to the vehicle injection. S100-β was elevated in the 3 and 7 days BMP7-injected PLX mice as well as in the 7 days BMP7-injected control mice, compared to the respective vehicle controls. TXNIP levels did not change in the control and PLX mice injected with vehicle or BMP7 3 days postinjection. Seven days postinjection, TXNIP levels did increase in the control BMP-injected mice, while no such change was observed in the PLX mice. No statistical significance was observed in the densitometric analysis (B) of blots from (A). (TIF 472 kb)

## Abbreviations

BMP: Bone morphogenetic protein
CCL: Chemokine ligand
CD: Cluster of differentiation
CSF: Colony stimulating factor
EGFR: Epidermal growth factor receptor
GFAP: Glial fibrillary acidic protein
GM-CSF: Granulocyte macrophage colony stimulating factor
GS: Glutamine synthetase
IFN: Interferon
IL: Interleukin
LCN: Lipocalin
MMP: Matrix metalloproteinases
NCAN: Neurocan
PCAN: Phosphacan
TAK: TGF-β activated kinase
THBS: Thrombospondin
TLR: Toll like receptor
TNF: Tumor necrosis factor
TXNIP: Thioredoxin interacting protein
VEGF: Vascular endothelial growth factor
VIM: Vimentin
