# Supplementary figures and images for: Microglia activation is essential for BMP7-mediated retinal reactive gliosis
Source: J Neuroinflammation. 2017 Apr 5;14:76. doi: 10.1186/s12974-017-0855-0 (PMC5382432; doi:10.1186/s12974-017-0855-0)

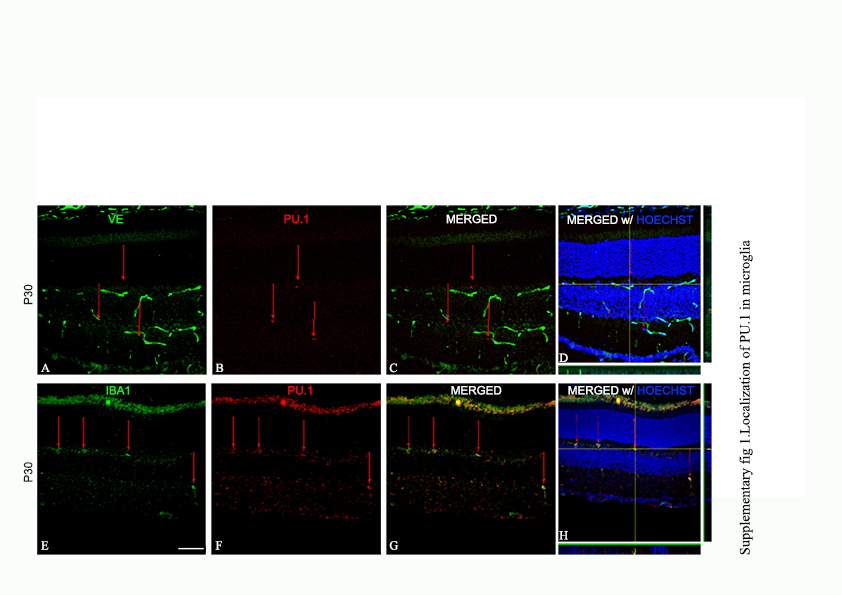

Supplement: Supplementary file 1 — PU.1 localizes with retinal microglia. Co-label of PU.1 antibody with antibody against GFP that cross-reacts with YFP in a retinal section from P30 mice which have YFP tag on vascular endothelial cadherin (VE-YFP), a marker expressed in endothelial cells (A-D). No co-label of PU.1 was observed with VE-YFP. PU.1 was also co-labeled with microglia marker IBA1 to show localization was restricted to microglial cells (E-H). Hoechst merged with the images of green and red channels are shown in D and H. Magnification bar in E = 50 μm, for images A–H. (TIF 857 kb) [file 12974_2017_855_MOESM1_ESM.tif]

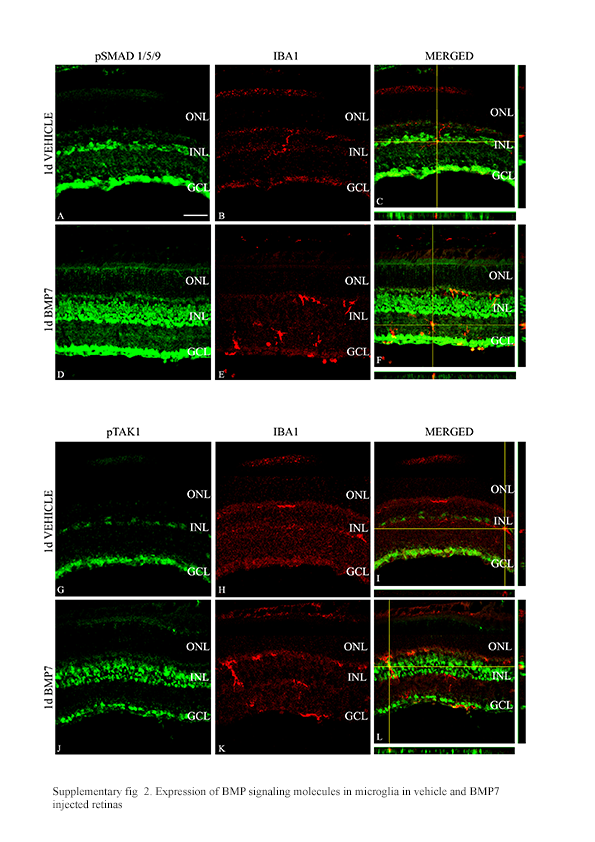

Supplement: Supplementary file 2 — Expression of BMP signaling molecules in microglia in vehicle and BMP7-injected retinas. Retinal sections from P30 mouse injected with vehicle or BMP7 24 h postinjection were double-labeled with antibodies that labels microglia cytoplasm (IBA1) and phospho SMAD 1/5/9 (pSMAD; A–F) or phospho TAK1 (pTAK1; G–L). Thin plane confocal microscopy images with y,z (strips to right of the panel) and x,z planes (strips at the bottom of the panels) shown in C, F, I and L. pSMAD-labeled cells were primarily found in the GCL in the vehicle-treated retina, with some co-localization with the cytoplasmic microglial marker IBA1 (A-C). The BMP7-injected retina had an increase in pSMAD expression in the INL as well as substantial co-localization with IBA1 (D–F). Vehicle-injected retina showed pTAK1 expression in the GCL with little to no IBA1 co-localization (G–I), while the BMP7-injected retinas showed increased levels of pTAK1 levels in the INL, as well as significant co-localization with IBA1 (J–L). Magnification bar in A = 50 μm, for images A–L. (TIF 688 kb) [file 12974_2017_855_MOESM2_ESM.tif]

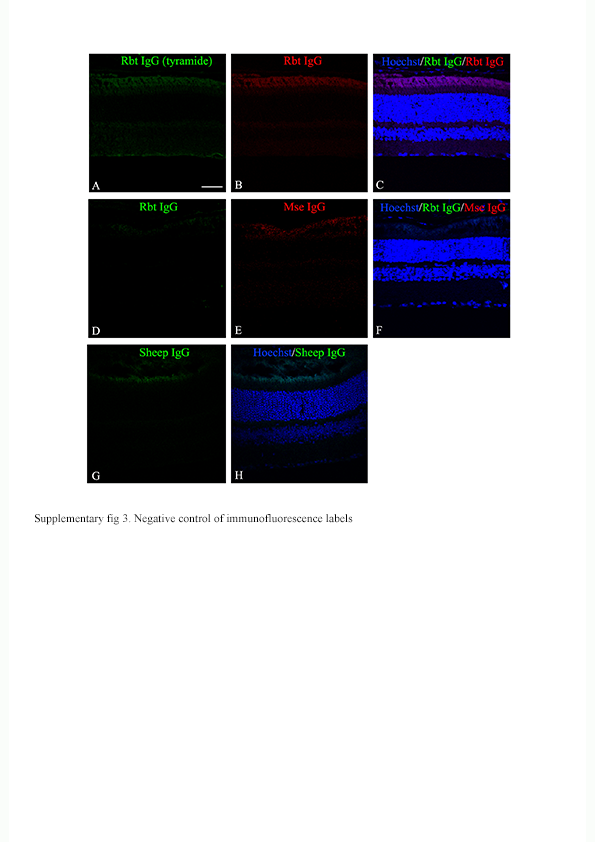

Supplement: Supplementary file 3 — Negative control of immunofluorescence labels. Retinal sections from P30 mouse labeled with rabbit immunoglobulin G (Rbt IgG; A–C, D, F), mouse IgG (Mse IgG; E, F), and sheep IgG (G, H) to determine background fluorescence. Images of sections labeled with the nuclear stain, Hoechst merged with the images of green and red channels are shown in C and F. Panels A–C represent sections, which were labeled with IgG following the procedure used for tyramide amplification when using two antibodies for the same species. Images in D–F represent sections co-labeled with rabbit and mouse IgG. Images A–C are negative controls for Fig. 1 and Additional file 1: Figure S1. Images D–F are negative controls for sections labeled with GFAP, S100-β, Calbindin, Brn3a, Chx10, Sox9, and IBA1. Images G and H are negative control sections for NCAN-labeled slides. Magnification bar in A = 50 μm, for images A–H. (TIF 465 kb) [file 12974_2017_855_MOESM3_ESM.tif]

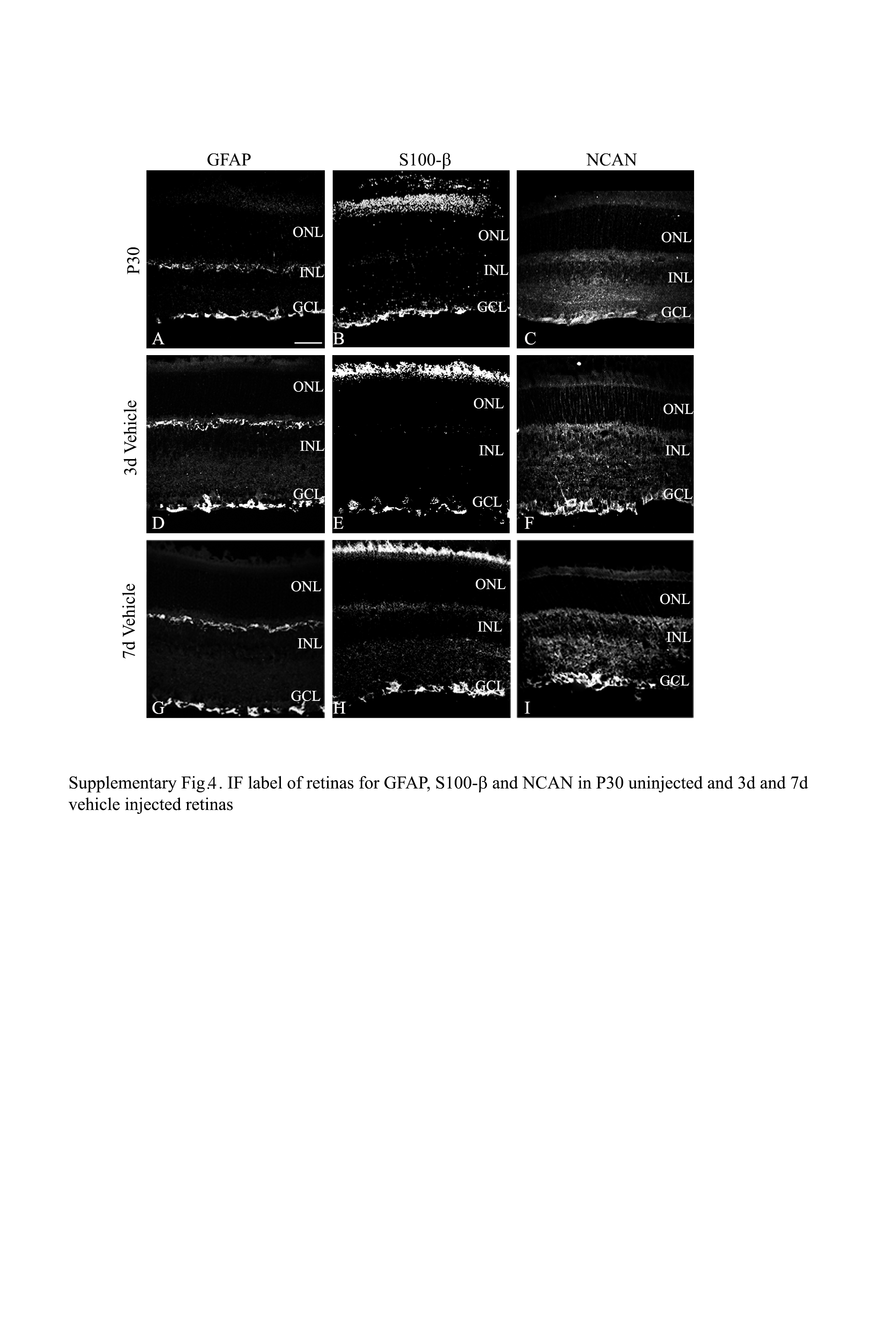

Supplement: Supplementary file 4 — IF label of retinas for GFAP, S-100-β, and NCAN in P30 uninjected and 3 and 7 days vehicle-injected retinas. Retinal sections from uninjected P30 mouse, vehicle-injected P30 mouse, obtained 3 and 7 days postinjection, labeled for GFAP (A, D, G), S100-β (B, E, H), and NCAN (C, F, I). Label for all three markers appears to be similar in the uninjected and the vehicle-injected retinas. Magnification bar in A = 50 μm, for images A–I. (TIF 5611 kb) [file 12974_2017_855_MOESM4_ESM.tif]

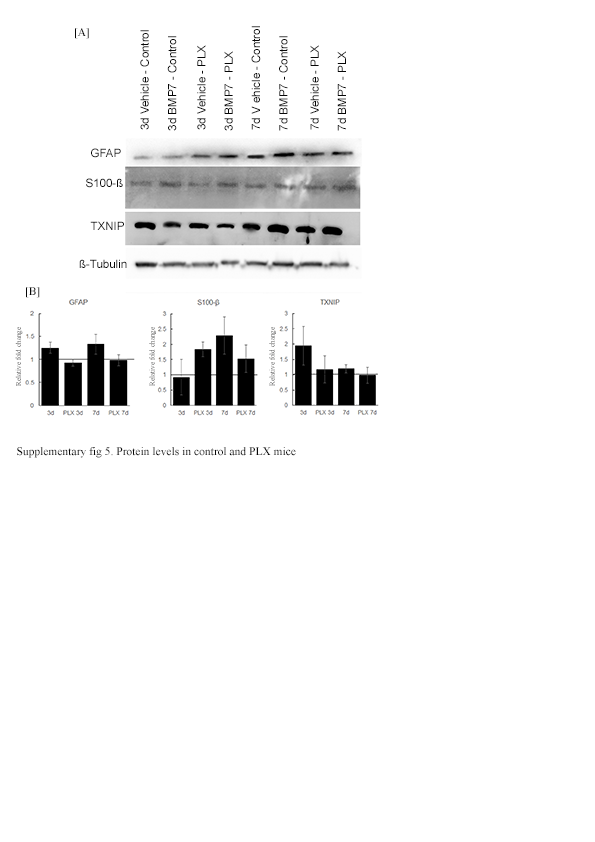

Supplement: Supplementary file 5 — Protein levels in PLX-treated mice. Protein isolated from control and PLX-treated mice injected with vehicle or BMP7 changes in protein levels of gliosis markers GFAP, S100-β, and TXNIP, with β-Tubulin used as a loading control. GFAP showed elevated levels in the BMP7-injected control mice, while PLX mice had GFAP levels similar to the vehicle injection. S100-β was elevated in the 3 and 7 days BMP7-injected PLX mice as well as in the 7 days BMP7-injected control mice, compared to the respective vehicle controls. TXNIP levels did not change in the control and PLX mice injected with vehicle or BMP7 3 days postinjection. Seven days postinjection, TXNIP levels did increase in the control BMP-injected mice, while no such change was observed in the PLX mice. No statistical significance was observed in the densitometric analysis (B) of blots from (A). (TIF 472 kb) [file 12974_2017_855_MOESM5_ESM.tif]
